# Transformation of Mycosis Fungoides/Sezary Syndrome: Clinical Characteristics and Prognosis

**DOI:** 10.4274/tjh.2016.0502

**Published:** 2018-03-06

**Authors:** Seçil Vural, Bengü Nisa Akay, Ayşenur Botsalı, Erden Atilla, Nehir Parlak, Aylin Okçu Heper, Hatice Şanlı

**Affiliations:** 1 Ankara University Faculty of Medicine, Department of Dermatology, Ankara, Turkey; 2 Ankara University Faculty of Medicine, Department of Hematology, Ankara, Turkey; 3 Etimesgut Şehit Sait Ertürk State Hospital, Clinic of Dermatology, Ankara, Turkey; 4 Ankara University Faculty of Medicine, Department of Pathology, Ankara, Turkey

**Keywords:** Anaplastic, Transformation, Mycosis fungoides, Transformed, Allogeneic hematopoietic stem cell transplantation, Sezary syndrome

## Abstract

**Objective:**

Transformed mycosis fungoides (T-MF) is a rare variant of MF with an aggressive course. In this study, we aimed to describe characteristics of MF/Sezary syndrome (SS) patients with transformation.

**Materials and Methods:**

Patients diagnosed with T-MF among MF/SS patients between 2000 and 2014 in a tertiary single center were evaluated retrospectively. Demographic data, clinical data, laboratory data, immunophenotype features, response to treatment, survival, and histopathologic features were analyzed.

**Results:**

Among 254 MF patients, 25 patients with T-MF were identified (10.2%) and included in the study. The male-to-female ratio was 2.6/1. The median time between MF diagnosis and transformation was 32 months (range: 0-192). Nine (36%) patients were diagnosed initially with T-MF. Advanced disease stage and high serum lactate dehydrogenase (LDH) levels were indicators of poor prognosis and treatment response. Five of the 18 patients with progressive disease had undergone allogeneic hematopoietic stem cell transplantation (allo-HSCT). Allo-HSCT resulted in complete remission in three (60%) patients. Ten (40%) patients died as a result of disease progression. Mean survival time was 25.2±14.9 (2-56) months after transformation.

**Conclusion:**

Advanced stage, high serum LDH levels, and loss of CD26 and CD7 expression in the peripheral blood are poor rognostic factors in T-MF. Treatment-resistant tumors and nodules should be cautionary for T-MF. Patients with T-MF have a shortened survival. Some patients may respond to first-line treatments. However, the majority of patients who do not respond to first-line therapies also are unresponsive to second or third-line therapies. Allo-HSCT may be an alternative option in patients with T-MF.

## Abstract (tr)

**Amaç:**

Transforme mikozis fungoides (T-MF) MF nadir görülen agresif seyirli bir alt tipidir. Bu çalışmada transformasyon gelişen MF/Sezary sendromu (SS) hastalarının klinik ve laboratuvar özelliklerinin değerlendirilmesi amaçlanmıştır.

**Gereç ve Yöntemler:**

Bu çalışmada tek bir referans merkezde 2000-2014 yılları arasında takip edilen MF/SS hastaları arasından T-MF geliştirenler retrospektif olarak değerlendirilmiştir. Demografik, klinik ve laboratuvar veriler, immünfenotiplendirme, tedavi yanıtları, histopatolojik özellikler ve sağkalım analiz edilmiştir.

**Bulgular:**

Takip edilen 254 MF hastası içerisinde 25 T-MF saptanarak (%10,2) çalışmaya dahil edilmiştir. Erkek kadın oranı 2,6/1’dir. MF tanısı ile T-MF tanısı arasında geçen sürenin medianı 32 ay olarak tespit edilmiştir (0-192). Dokuz hastada (%36) tanı anında transformasyon bulunmaktadır. İleri hastalık evresi ve yüksek serum laktat dehidrogenaz (LDH) düzeyleri kötü prognoz ve tedavi yanıtı göstergesi olarak saptanmıştır. Tedaviye dirençli 18 ileri evre hastadan beşine allojenik hematopoetik kök hücre transplantasyonu (allo-HKHT) yapılmıştır. Bunlardan üçünde tam remisyon sağlanmıştır. İzlemde toplam 10 hasta hastalık progresyonu nedeniyle kaybedilmiştir. T-MF sonrası ortalama sağkalım 25,2±14,9 (2-56) aydır.

**Sonuç:**

İleri hastalık evresi, yüksek LDH düzeyi, perifer kan T hücrelerde CD26 ve CD7 kaybı kötü prognoz belirteçlerindendir. Tedaviye dirençli nodül ve tümörler T-MF açısından şüphe uyandırmalıdır. T-MF’de sağkalım kısalmıştır. Bazı hastalarda birinci basamak tedavilere iyi yanıt alınabilmektedir. Ancak birinci basamak tedavilere yanıtsız hastalar genellikle ikinci ve üçüncü basamak tedavilere de direnç gösterebilmektedir. Allo-HKHT, T-MF hastalarında alternatif bir tedavi yöntemi olarak kullanılabilir.

## Introduction

Mycosis fungoides (MF) is the most common subtype of cutaneous T-cell lymphoma (CTCL). Generally, MF has an indolent course with slow progression from patch/plaque-stage disease to cutaneous tumors [1]. However, in the case of large-cell transformation (LCT), it is associated with an aggressive clinical course and poor survival [2].

Diagnosis of transformed MF (T-MF) is based on the presence of large cells (CD30 +/-) exceeding 25% of the infiltrate throughout the lesion or forming microscopic nodules of large cells[3]. Molecular studies have demonstrated that the large-cell infiltrate in T-MF/Sezary syndrome (SS) represents evolution from the original clone [4].

Advanced stage of MF at the time of transformation and folliculotropism are suggested as the most important factors affecting survival[2]. Additionally, early transformation in MF lesions was described as a poor prognostic factor in previous studies [5]. Even though the CD30 expression is more common in advanced MF, in T-MF, it is reported as a favorable prognostic factor [6,7,8].

Risk factors associated with an aggressive course of T-MF are not well described in the literature due to the low incidence of MF/SS and thus T-MF. In different series, the incidence of T-MF has been reported to range between 8% and 55%among MF patients [3,5,9,10,11]. This study was designed to investigate the clinical, laboratory, and histopathological parameters associated with T-MF.

## Materials and Methods

We retrospectively evaluated all MF/SS patient records in a single reference center in Ankara, Turkey, from 2000 to 2014. Among all MF/SS patients, T-MF patients with at least one histopathologically confirmed biopsy were included in the study.

For each case, clinical features were evaluated by three dermatologists and histopathological findings were reviewed by one pathologist who was an expert in this area.

All patients were classified according to the International Society for Cutaneous Lymphomas and European Organisation of Research and Treatment of Cancer revised criteria of 2007 [12]. Staging included physical examination, blood cell count and chemistry, peripheral blood smear and flow cytometry, lymph node ultrasonography, and, in most cases, computed tomography scans of the abdomen, chest, and pelvis. Histopathology included one or multiple skin biopsies for all patients. In the case of clinically and sonographically significant adenopathy, a lymph node biopsy was performed.

The accompanying prognostic factors were also analyzed: age, sex, age at diagnosis of T-MF, presence of folliculotropism in skin lesions, CD30 expression in more than 75% of cutaneous neoplastic T cells, serum lactate dehydrogenase (LDH) levels, serum b2-microglobulin levels, and eosinophilia. The time interval between MF and T-MF, clinical stage at the time of T-MF, and survival were analyzed.

Therapies were classified as first-, second-, and third-line treatments according to the 2014 National Comprehensive Cancer Network Clinical Practice Guidelines in Oncology [13,14]. Allogeneic hematopoietic stem cell transplantation (allo-HSCT) and autologous stem cell transplantation were evaluated separately.

Response to treatment was evaluated as follows: complete response (CR), complete resolution of the disease; partial response (PR), at least 50% improvement compared with baseline; stable disease (SD), some improvement (25% to 50% improvement in lesions) plus reduction in the size of axillary and inguinal lymph nodes in the absence of significant evidence of disease; or progressive disease (PD), more than 25% increase in the number or size of clinically abnormal lymph nodes, or development of novel tumors or pathologically positive nodes or visceral disease [12].

### Statistical Analysis

The data obtained from patients were analyzed with SPSS 16.0. The Mann-Whitney U test, chi-square test, Spearman’s test, and Mantel-Cox analysis were used to compare variables. The Kaplan-Meier method was used to determine overall survival.

## Results

### Clinical Data

The disease stage and exact TNMB stages of patients, initial treatments, and follow-up data of each patient are summarized in Table 1. Durations between the diagnosis of MF and transformation and the follow-up duration are given in Table 2. The rate of T-MF was 10.2% (n=25) among all MF/SS patients (n=254). The median age at the time of MF diagnosis was 49 years (range: 26-76), whereas the median age at the time of transformation was 54 years (range: 30-78). The male-to-female ratio was 2.6 (M/F: 18/7). Sex was not significantly related to survival (p=0.218). Patients’ age at the time of transformation was also not related to survival (p=0.697). Transformation was detected in 36% (n=9) of the patients at the onset of MF. The median time between the diagnosis of MF and transformation was 32 months (range: 0-192). Patients were followed for a mean of 39.4±17.1 months after transformation.

**Figure 1:**
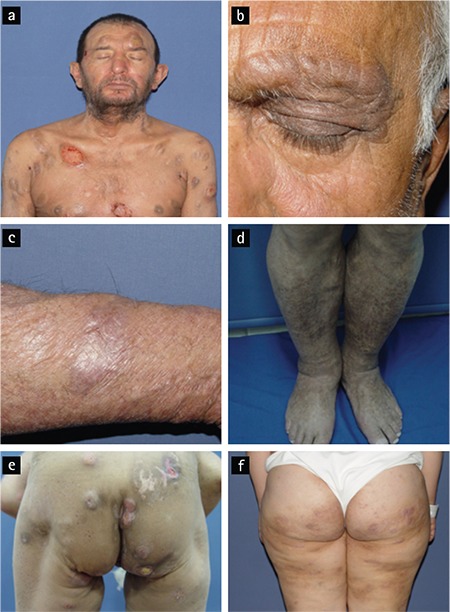
Histopathologically confirmed transformed mycosis fungoides (T-MF) lesions in different patients: extensive tumoral lesions with anaplastic transformation on the trunk (a); resistant tumoral lesion with loss of hair on eyebrow (b); refractory plaque on forearm (c); erythrodermic patient with ichthyotic lesions on legs, consistent with T-MF (d); postinflammatory hypopigmentary areas from previous treated tumors and tumoral lesions with anaplastic transformation (e); plaques and tumoral lesions on gluteal region and legs of a patient receiving extracorporeal photopheresis, interferon psoralen ultraviolet A, and bexarotene (f).

Two of 25 patients with T-MF (8%) had early patch and plaque MF (stage IA: 1, stage 1B: 1). Twenty-three patients had advanced-stage disease [stage IIB (n=9, 36%), stage III (n=3, 12%), stage IVA_1 _(n=8, 32%), stage IVA_2 _(n=2, 8%), and stage IVB (n=1, 4%)]. Most patients had transformation only at a skin site (96%); in one patient skin and lymph, node transformations were detected simultaneously (4%). Furthermore, 32% of T-MF patients presented with a new or enlarging tumor accompanied by long-standing plaque lesions. Dermatological examination at the time of T-MF diagnosis for the rest of the patients revealed the following: two (8%) patients had long-standing enlarging tumors, four (16%) patients had a new tumor accompanied with erythroderma (one bullous), one (4%) patient demonstrated ichthyosiform erythroderma, three (12%) patients had an enlarging plaque with erythroderma, three (12%) patients had long-standing plaques, two (8%) patients had newly scattered papules distinct from MF plaques, one (4%) patient showed an abrupt onset of multiple pink scattered nodules, and another patient (4%) had follicular papules associated with hair loss within the involved area (Figure 1).

Histopathological examinations of the transformation site showed tumoral lesions in 18 (72%) cases and plaque lesions in seven (28%) cases. Lesion subtype (plaque or tumor) was not significantly correlated with survival (p=0.678). Less prominent or focal epidermotropism was present in 15 (60%) of the 25 patients in our study, and only 2 (8%) patients had Pautrier microabscesses. Folliculotropism was observed in ten cases (40%) with LCT. In eight (80%) of them, there was progression under treatment, while in one (10%) patient PR to treatment and in 1 (10%) patient CR was observed. Folliculotropism was not correlated statistically with survival (p=0.568).

Immunophenotype analysis of the skin biopsies showed that 24 (96%) patients had a CD3^+^CD4^+^CD8^− ^T-cell phenotype and one (4%) patient had a double CD4^+^CD8^+ ^T-cell aberrant phenotype. In most cases (88%), there was partial loss of one or more pan-T-cell antigens. Loss of CD7 expression was seen in 22 (88%) patients. 

CD30 positivity in more than 75% of all the large T cells was present in skin biopsies of five patients (20%). In the remaining 20 (80%) cases, CD30 staining was either completely negative or expressed by only a very few (<5%) large T cells. There was no statistically significant difference either in disease stage or treatment response among CD30-positive and CD30-negative patients (p=0.290, p=0.630). Twelve patients with early (<2 years, n=3, 12%) and concurrent (n=9, 36%) transformation were also evaluated separately for survival (p=0.582).

**Figure 2:**
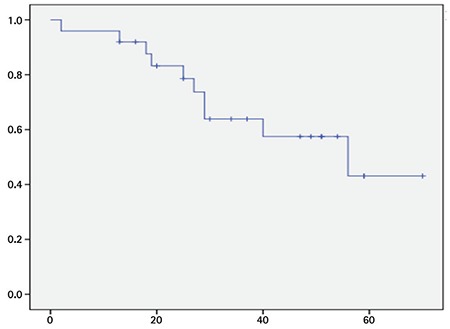
Kaplan-Meier survival curve: survival in months after anaplastic transformation.

Advanced disease stage at the time of transformation correlated with poor survival (p=0.003). In our study, among the deceased patients, 80% had stage IV disease, whereas only 20% of patients had stage IV disease among the surviving patients (p=0.002). During follow-up, 10 patients died of disease-related events (32%). Three (30%) of 10 patients who died in our study had SS, and the other patients’ stages were as follows: stage IIB (n=1, 10%), stage III (n=1, 10%), and stage IVA (n=5, 50%). The survival curve of the patients is presented in Figure 2. Mean survival time was 25.2±14.9 (2-56) months after transformation.

### Laboratory Findings

Flow cytometry of peripheral blood showed an increased ratio of CD4/CD8 (>2) in 13 (52%) patients. The ratio was between 2 and 10 in ten (40%) patients and higher than 10 in three (12%) patients. The patients’ disease stages and CD4/CD8 levels showed a statistically significant positive correlation (p=0.038). Increased CD4+/CD26 cell ratio was significantly correlated with poor survival (p=0.017). Loss of CD7 expression (more than 40%) was significantly related to poor survival (p=0.001).

Laboratory findings are summarized in Table 3. High serum LDH levels were correlated significantly with poor survival (p=0.000). There was a statistically significant relationship between elevated serum LDH levels and advanced disease stage (p=0.028). The disease stage and b2-microglobulin levels were found to be positively correlated with Spearman’s test (p=0.026, r=0.463). There was no statistically significant relationship between b2-microglobulin levels and survival (p=0.125).

### Treatment

All patients received first-line therapy as a combination treatment of two or more of the following: psoralen plus ultraviolet, interferon-alpha, extracorporeal photopheresis, vorinostat, bexarotene, retinoid, low-dose methotrexate, local radiotherapy, or total skin electron beam radiotherapy. Of the 18 (72%) patients showing PD with first-line treatment modalities, 12 (48%) patients received either second- or third-line treatments. Of these 12 patients, six (48%) received second-line treatments either for the induction of remission or in an attempt to decrease tumor burden before allo-HSCT. Second-line therapy included single-agent chemotherapy of either gemcitabine or pralatrexate in 5 (20%) patients to decrease the tumor burden before allo-HSCT. One patient had a lymph node biopsy consistent with concomitant natural killer cell lymphoma and received an Aurora A kinase inhibitor as second-line therapy following five cycles of multiagent chemotherapy. All of the patients’ treatment responses with second-line treatment were evaluated as PD.

Seven (28%) patients received third-line therapy due to PD. Additionally, three (12%) patients had received multiagent chemotherapy before first- and second-line treatments before admission to our center. In all patients, the treatment responses of the third-line treatments were evaluated as PD.

Five (20%) patients with PD underwent allo-HSCT and CR was achieved in 3 (60%) of them after the procedure. Two patients’ disease recurred 2 and three months after allo-HSCT, and these two patients died 9 and 11 months following transplantation, respectively. One patient in follow-up with complete remission died 24 months after allo-HSCT due to sepsis. Autologous stem cell transplantation was performed in one patient in 2000, and the patient died four months after the procedure due to disease progression. The outcome of patients with HSCT is given in Table 4.

## Discussion

LCT of MF can occur at any stage of MF, and it has been associated with disease progression and poor outcome. Unfavorable prognostic factors for T-MF have been reported previously as advanced stage, presentation of MF with transformation, generalized skin tumors, increased LDH level, and use of combination chemotherapy [5,15]. CD30 expression in less than 10% of skin lesions is one of the poor prognostic factors [5,6,8,15]. On the other hand, in this study, high serum LDH levels, loss of CD26 expression of more than 30% in peripheral blood, and loss of CD7 expression were associated with poor survival among T-MF patients.

LCT at initial diagnosis of MF or within two years has been associated with worse prognosis in several studies [3,5,16,17,18]. However, in some studies, including ours, the prognostic significance of early transformation was not validated [19]. Mean time between MF and T-MF diagnosis varies from 44 months to 6.5 years in reported studies [2,7,17,19]. In our study, this period was determined as 32 months.

In previous studies, LCT of MF has been reported mainly in advanced disease. In a series with 22 T-MF patients, Arulogun et al. [17] reported that only 1.4% of early-stage MF patients developed T-MF, whereas this rate was more than 25% in stage 2B patients and more than 50% in stage 4 patients. Consistent with this study, LCT of MF was detected in 8% of cases in the early stage in our series. MF is a slowly progressive CTCL with an excellent prognosis, especially in early-stage disease. In the case of transformation, the prognosis of early-stage MF deteriorates significantly [20]. Still, previous studies have shown that patients with early (stage I-IIA) LCT have longer survival compared with patients with LCT in advanced disease (stage IIB-IV) [5]. Likewise, in our study, advanced disease stage at the time of transformation was significantly correlated with shorter survival. In different studies, extracutaneous disease (stage IV) was shown to be associated with poor prognosis [2,19]. In our study, a significant majority of the patients who died had stage IV MF. The mean survival time after LCT has been reported to be in the range of 2 to 36 months [2,3,4,5,7,9,10,17,19]. In our study, the mean survival time of the ten patients who died was determined as 25.2±14.9 (2-56) months.

Transformed folliculotropic MF patients were previously found to have shorter survival time [2,21,22]. In one previous study, epidermotropism was detected in patients with LCT, although it was less prominent or focal [3]. In our series epidermotropism was present only in 60% of patients and it was less noticeable in histopathological examinations.

According to a recent study, several clinical characteristics such as a new solitary nodule on MF plaques or rapidly presenting scattered papules may be indicators of the development of LCT for dermatologists [15]. We would like to emphasize that transformation was also observed in treatment-refractory long-standing tumoral and plaque lesions, and in a patient with ichthyosiform erythroderma. For this reason, in addition to the cautionary skin findings mentioned before, reevaluation of treatment-resistant and unexpected lesions, especially in advanced-stage patients, is recommended.

The treatment strategy is challenging in T-MF. It is important to note that, among our patients with advanced stages of T-MF, none had a CR to treatment under first-line therapies. Among patients receiving first-line therapy, 20% had either SD or PR with these therapies. Notably, all the patients who received second- and third-line therapies had PD. This finding highlights the refractory nature of T-MF. In fact, aggressive treatment strategies and multiple chemotherapies for MF result in a short period of CR, followed by an aggressive relapse [13]. Allo-HSCT is an emerging effective therapy in MF/SS, demonstrating a decrease in the relapse rate and an overall increase in disease-free survival. It was reported that one year after allo-HSCT, 42% of patients remained in remission [23]. In our series, 60% of patients were in remission one year after transplantation. Transplant-related mortality and infections are significant factors decreasing the success rate of allo-HSCT. However, in selected patients with T-MF, allo-HSCT increases disease-free survival and thus the quality of life.

### Study Limitations

A limitation of the present study was the small number of patients with T-MF, highlighting the rarity of MF/SS. A second limitation was the retrospective design of the study, which may have restricted retrieval of the data from patient archives.

## Conclusion

Unfavorable prognostic factors in T-MF include advanced stage, high serum LDH levels, and loss of CD7 and CD26 expression in T helper cells. In patients with treatment-refractory tumors and unusual lesions, a biopsy is warranted to exclude T-MF. Patients with T-MF have a short life expectancy. Patients may have CR, PR, or SD with first-line treatments, which underlines the value of less aggressive therapies. However, nonresponders usually do not respond to second- or third-line therapies. Allo-HSCT may be an alternative option for patients with T-MF.

## Figures and Tables

**Table 1:**
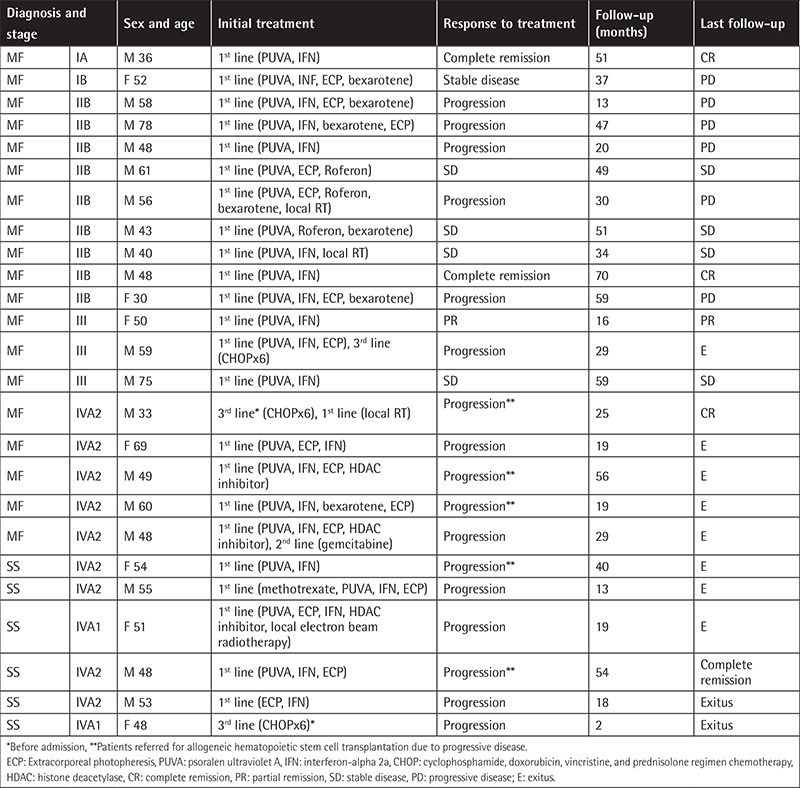
Characteristics of patients with transformed mycosis fungoides.

**Table 2:**
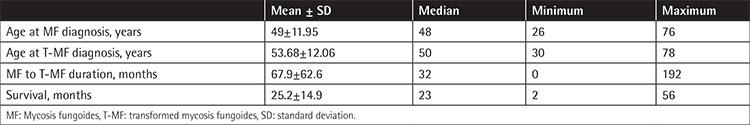
Clinical features of transformed mycosis fungoides patients.

**Table 3:**
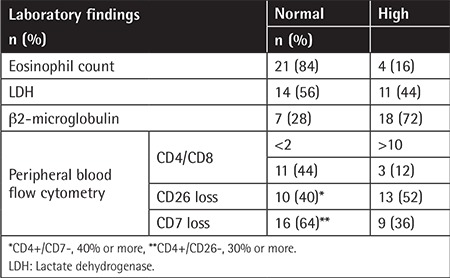
Laboratory findings of transformed mycosis fungoides patients.

**Table 4:**
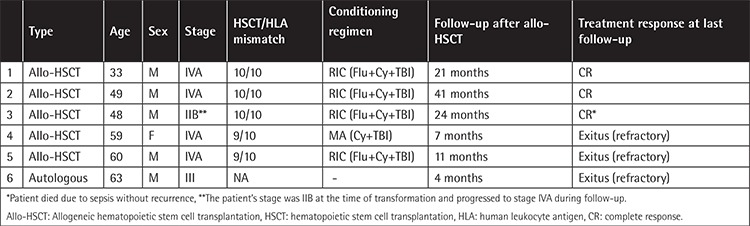
Clinical features and treatment results of the patients who had undergone allogeneic hematopoietic stem cell transplantation.
